# Comprehensive comparison of salivary gland and cutaneous adnexal tumors: analogous versus discrepant clinical, terminological, histological, and molecular features: Part II: Second series of related tumor entities and synoptic comparison of general aspects

**DOI:** 10.1007/s00428-026-04397-2

**Published:** 2026-01-23

**Authors:** Stephan Ihrler, Lukas Greber, Abbas Agaimy, Christian Haas, Philipp Jurmeister, Almut Böer-Auer

**Affiliations:** 1 Institute of Pathology, Ludwig-Maximilians-University, Munich, Germany; 2 DERMPATH Muenchen, Bayerstrasse 36, Munich, D-80335 Germany; 3 Department of Oral and Plastic Maxillofacial Surgery, Military Hospital, Ulm, Germany; 4 Pathological Institute, Friedrich-Alexander University Erlangen, University Hospital, Erlangen, Germany; 5 Dermatologikum Hamburg, Hamburg, Germany; 6 Department of Dermatology, Münster University, Münster, Germany

**Keywords:** Salivary gland, Cutaneous adnexal, Tumor, Histogenesis, Molecular pathology, Terminology

## Abstract

This publication presents in two parts a comprehensive comparison of the manyfold salivary gland and cutaneous adnexal tumors, investigating both striking similarities and discrepancies with respect to clinical aspects, terminology, (immune)-histology, and molecular pathology. Part I has presented basic embryology/histology and overlapping topographical aspects, followed by a first series of related tumor entities. In this second part, a further series of histologically analogous/similar tumor entities are compared, comprising secretory and microsecretory carcinoma, sialadenoma and syringocystadenoma papilliferum, adenoidcystic carcinoma, mucinous and mucoepidermoid tumors, lymphoepithelial tumors, sebaceous tumors, and further rare related tumor groups. Finally, in a synoptic comparison, general features are summarized: While tumors with follicular differentiation are lacking in salivary glands, tumors with acinar and oncocytic differentiation are lacking in the skin. In general, salivary tumors have a higher proportion of malignancy, are on average larger, are frequently more difficult to resect due to a more complex topography, and, hence, show a higher propensity for recurrence and metastases, on average with a poorer prognosis. Multifocality with or without link to hereditary tumor syndromes is not infrequent in cutaneous lesions, but is very rare in salivary tumors.

## Introduction

The manyfold salivary gland (*n* = 36) and cutaneous adnexal (*n* = 47) tumor entities according to WHO classifications [1, 2] exhibit a bewildering mixture of striking similarities/analogies, but also major discrepancies. This publication in two parts presents a comprehensive comparison of similarities and discrepancies in clinical aspects, terminology, (immune)-histology, and molecular pathology.

Part I focused on a comparison of embryology and basic microscopic anatomy of both organs, followed by a discussion of diagnostically relevant topographical overlaps, especially in periparotid and perioral locations [3], and by a first series of related tumor entities. In the herein presented part II, a further series of histologically analogous or similar entities are compared, starting with tumor entities, where almost all criteria are identical in salivary and cutaneous tumors: secretory and microsecretory adenocarcinoma and sialadenoma versus syringocystadenoma papilliferum. Thereafter, a series of related tumor pairs with partial overlap of the different features is presented: adenoid cystic carcinoma, mucinous and mucoepidermoid tumors, lymphoepithelial tumors, sebaceous tumors, and further rare pairs of tumors. Finally, a synoptic comparison will summarize major general features. Aspects of material and methods are identical as disclosed in part I.

## Secretory carcinoma

Secretory carcinoma is the prototypical example for those rare tumor entities, which are almost completely identical in salivary glands and the skin. The only difference is epidemiology, being relatively frequent in major and minor salivary glands, but rare in the skin. Secretory carcinoma had first been described in the breast several decades ago, then in 2009 in skin [4], and in 2010 in salivary glands [5].

**Fig. 1:**
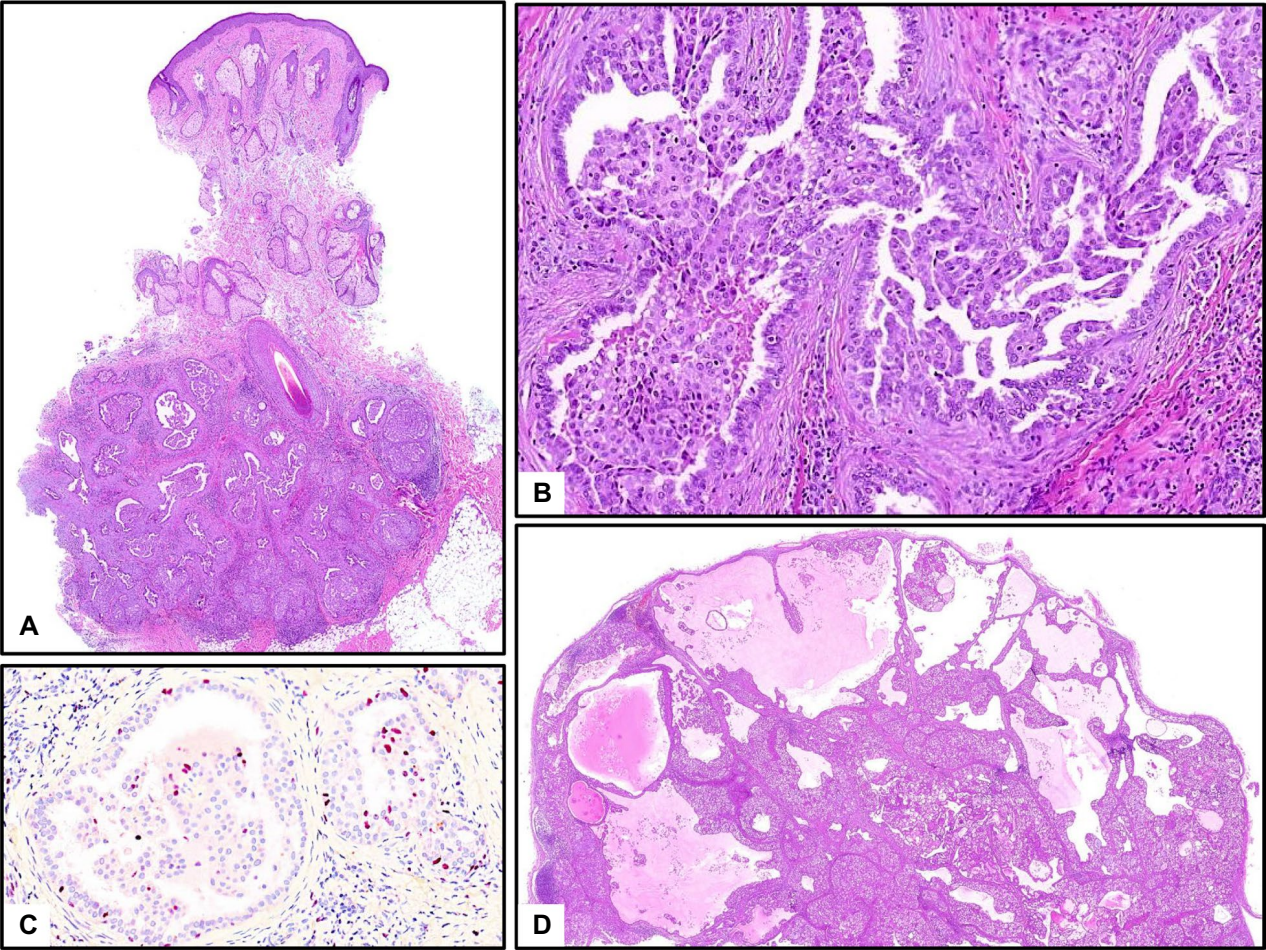
Cutaneous secretory carcinoma with *ETV6::NTRK3* translocation. **A** Overview with skin (top) and moderately circumscribed, partly cystic tumor in deeper dermis and subcutis. **B** Higher magnification of a cystic/micropapillary glandular proliferation with desmoplasia, without significant atypia. **C** Low proliferation with Ki67. **D** Ipsilateral cervical lymph node metastasis, manifesting five years later. abundant pink intracystic secretion (“secretory carcinoma”). A case of a salivary secretory carcinoma is shown in Fig. 4 in part I

It is histologically a mostly well-circumscribed, (micro-)cystic, partly solid glandular tumor with a variable amount of eosinophilic secretion, absent or minor cellular atypia, and slightly increased proliferation (Part I: Fig. 4). In the skin, it presents as a non-encapsulated nodule in the dermis and/or subcutis with no connection to the epidermis (Fig. 1), most commonly in the axilla [6]. Characteristic, but not specific immunoreactions (CK7, Gata3, mammaglobin, S100, and STAT5 positive, NTRK for therapeutic options, DOG-1 negative) enable distinction from the main differential diagnosis of acinar cell carcinoma (in salivary location).

Frequently, a recurrent and specific translocation, t (12;15) (p13;q25), is found, resulting in *ETV6::NTRK3* fusion and rarely RET fusions (in > 90% of cases), which are useful in difficult cases [5]. In both organs, it is a low-grade carcinoma with rare recurrences and metastases and a favorable prognosis. Figure 1 demonstrates the rare case of a cutaneous secretory carcinoma of the cheek with cervical lymph node metastasis (Fig. 1D).

## Microsecretory carcinoma

A similar constellation with completely identical criteria in salivary and cutaneous cases is with microsecretory carcinoma, described for the first time in 2018 in minor salivary glands [7] and in 2022 in the skin [8]. The tumors are typically small to medium-sized nodules, often with a minor degree of infiltration, with characteristic, isomorphic microcysts with unilayered epithelium, largely devoid of cellular atypia, and with low proliferation. The intraluminal secretion has a basophilic appearance. Immunohistochemistry shows expression of S100, SOX10, and variable p63, while Mammaglobin is not expressed (in contrast to secretory carcinoma) [9].

All reported salivary and cutaneous cases so far exhibited the recurrent and specific translocation *MEF2C:SS18*, an important diagnostic criterion in difficult cases [7–10]. Based on limited experience, the prognosis appears to be very favorable in both salivary glands and skin. Our group has published the first case (buccal salivary gland) with recurrence and metastasis (lung), supporting the categorization of this tumor entity as low-grade carcinoma [10].

## Sialadenoma papilliferum versus syringocystadenoma papilliferum

**Fig. 2:**
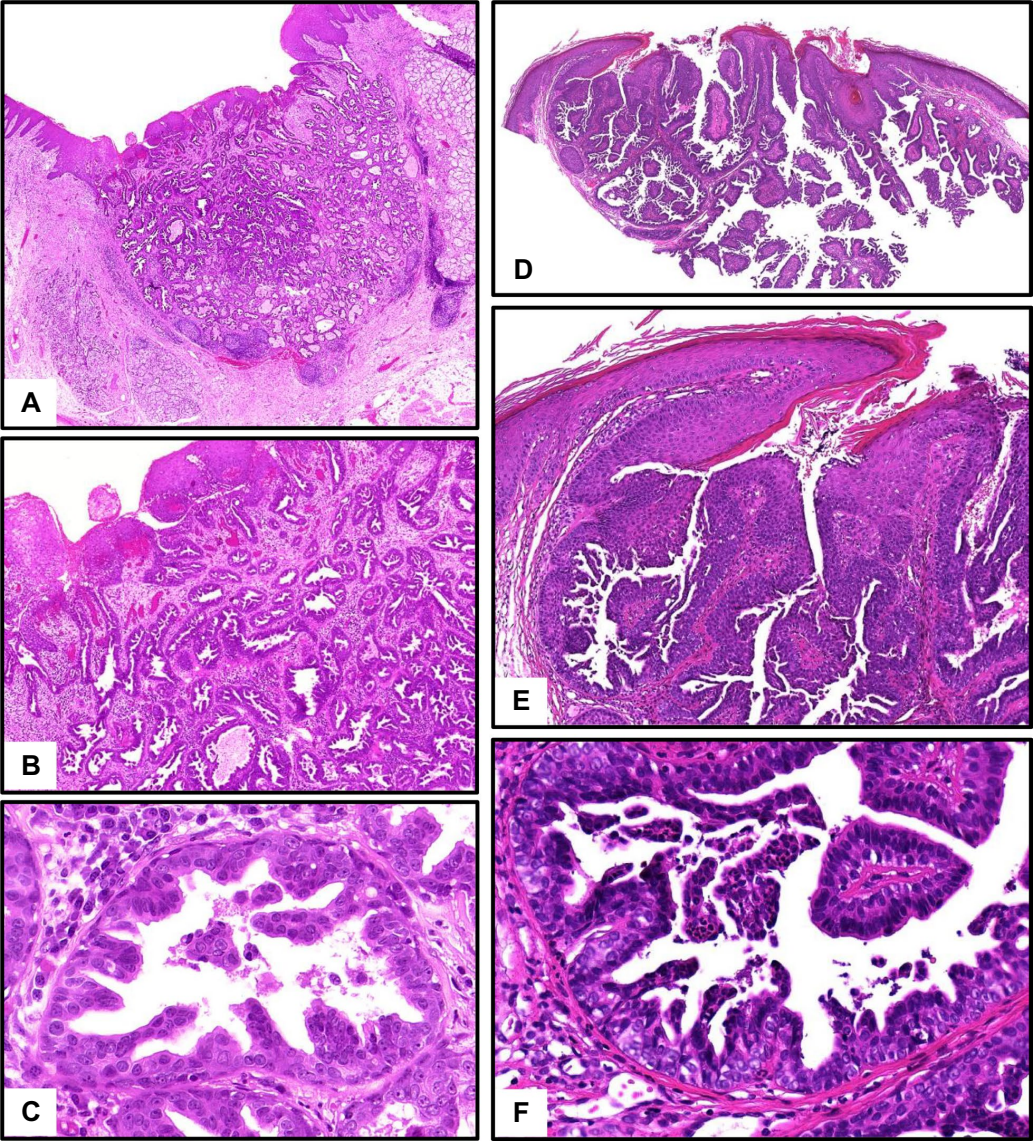
**A**–**C** Salivary sialadenoma papilliferum. **A** Well-circumscribed, glandular-cystic tumor at the palate with fusion with acanthotic squamous mucosa. **B**, **C** Microcysts with bilayered epithelium with micropapillary projections, devoid of cellular atypia. **D**–**F** Cutaneous syringocystadenoma papilliferum. **D** Well-circumscribed glandular-cystic tumor with broad connection to hyperplastic epidermis. **E**, **F** Bilayered epithelium with micropapillary projections, devoid of cellular atypia. Case **A**–**C** with mutation of *BRAF-V600E*, case **D**–**F** not tested

Both tumor types are rare, the terminology is analogous, and the (immune-) histological and molecular presentation is largely identical. Both show a well-circumscribed, glandular-cystic, partly micropapillary architecture with a bi-layered epithelium with basal cells (CK14, p63), devoid of atypia or increased proliferation, superficially with transition into hyperplastic squamous epithelium of the skin or mucosa (salivary tumors are restricted to mucosa-related minor glands, mostly at the palate; Fig. 2) [1, 11].

Identical *BRAF-V600E* mutations have frequently been proven in both tumor types. The salivary tumor develops de novo, while syringocystadenoma papilliferum commonly arises as a secondary neoplasm in longstanding nevus sebaceous [11].

## Adenoid cystic carcinoma

Adenoid cystic carcinoma (ACC) of both organs is characterized by identical terminology and very similar (immune-)histology and molecular alterations; however, there is a strikingly discrepant frequency and prognosis. ACC represents one of the most frequent salivary carcinoma entities but is rare in the skin, where lesions appear most commonly in the head and neck area, often in the external auditory canal and rarely on the trunk or genital sites [12].

**Fig. 3:**
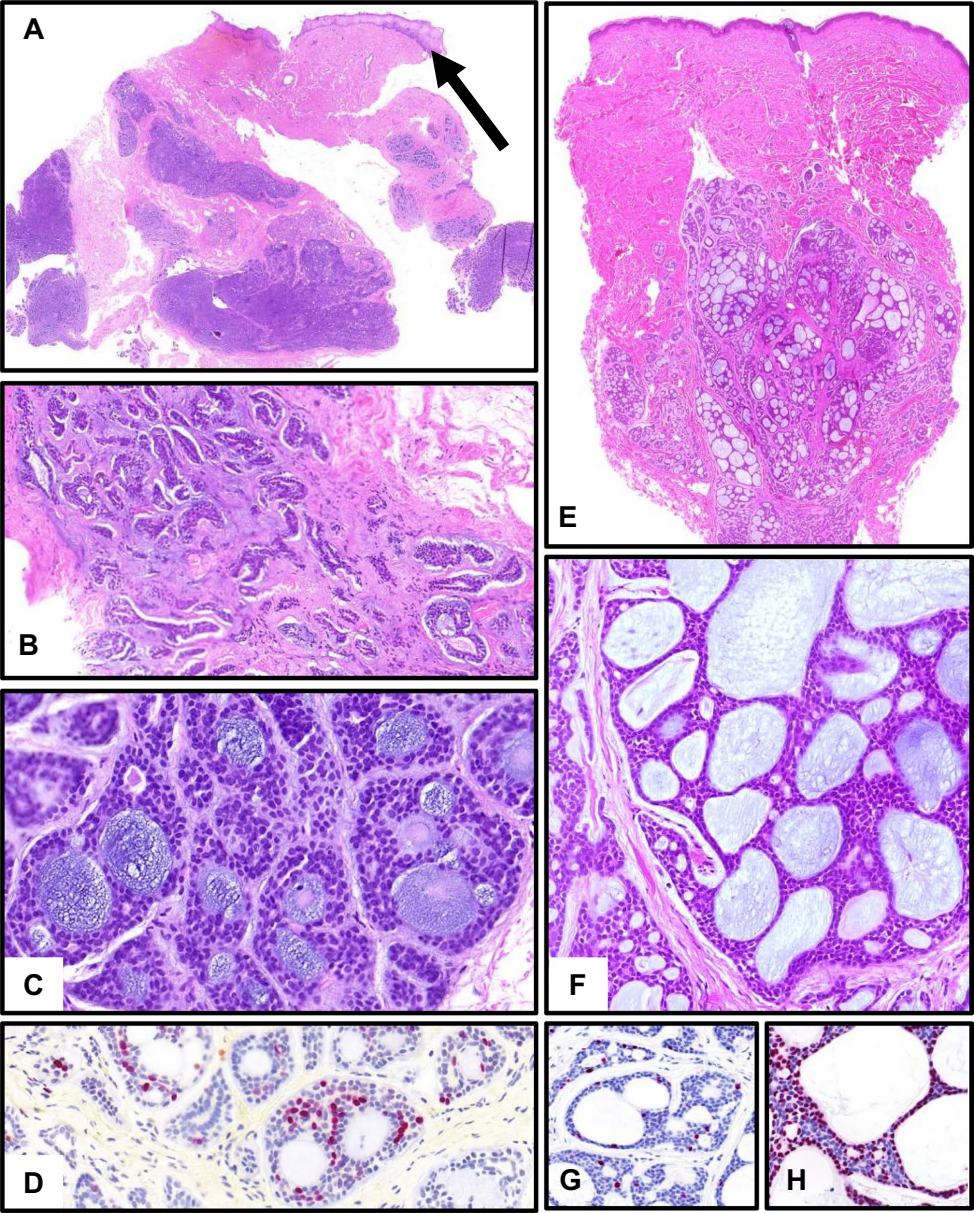
**A**–**D** Adenoid cystic carcinoma of minor salivary lip gland with translocation of *MYB::NFIB*. **A** Multiple nodules, obviously infiltrative, mucosa at the top (arrow). **B** Infiltrative tubular strands. **C** Bilayered epithelium with mixed tubular and cribriform architecture (with blue pseudo cysts). No relevant cellular atypia. **D** Low to moderate proliferation (Ki67). **E**–**H** Cutaneous adenoid cystic carcinoma on the scalp. **E** Infiltrative nodules in deep dermis and subcutis. **F** Preponderantly cribriform architecture with multiple blue pseudo cysts. No relevant cellular atypia. **G** Low proliferation (Ki67). **H** Biphasic differentiation, demonstrated by p63-positive abluminal myoepithelial cells

The histological hallmark is a biphasic luminal-abluminal (ductal-myoepithelial) differentiation with a variable admixture of usually dominating tubular, very characteristic cribriform (the so-called adenoid-cystic pattern), and less common solid structures (Fig. 3). Immunohistological expression of MYB may be helpful, while expression of CD117 is rather non-specific. The degree of cellular atypia, of proliferative/mitotic activity, and of infiltration varies in salivary ACC from low to pronounced, while cutaneous lesions show mostly bland cytology. Perineural invasion is frequent in salivary ACC and is rare in cutaneous cases. The diagnosis may be straightforward in resections, but can be very difficult in salivary biopsies (typically in minor glands) or cutaneous curettages, and generally in highly differentiated cases with absence of the characteristic cribriform structures. Histological grading of malignancy is not recommended; however, the so-called poorly differentiated, solid type of ACC with high-grade atypia and high proliferation, shows poor prognosis in salivary glands. High-grade transformation in cutaneous ACC is very rare [13]. Rearrangements of *MYB::NFIB* or, more rarely, *MYBL1::NFIB* are found in about 70% of salivary and cutaneous cases [2, 14].

Due to complex topography and frequently blunt invasion, complete surgical resection is problematic in advanced salivary ACC, rendering frequent local recurrences, late distant metastases, and fatal outcomes. Cutaneous ACC shows less infiltration and, hence, complete resection is easier, resulting in fewer recurrences and metastases and better prognosis [2]. Local recurrence in skin occurs after incomplete excision and when perineural growth is prominent. Metastases of cutaneous ACC to lymph nodes or distant sites (mostly lung) are rare, with increased risk in lesions in the external auditory canal and on the genitalia. Cutaneous metastasis of salivary ACC or of other sites (lacrimal gland, lung, mamma, etc.) is a very rare differential diagnosis and requires clinical information.

## The heterogeneous spectrum of mucinous/mucoepidermoid tumors

The heterogenous tumors harboring an obligate mucinous differentiation include three salivary and two cutaneous entities (Table 1). The rare benign salivary mucinous cystadenoma of mostly minor glands has no counterpart in skin. It is usually a small, uni- or multi-cystic glandular lesion with dominant mucinous differentiation, usually with a basal cell component (CK14, p63), lacking cellular atypia and invasion.

**Table 1:** Differential diagnosis of tumors with mucinous differentiation

Entity	CK14/p63	Estrogen receptor	Neuroendocrine markers	Extracellular mucin	Genetics	Relative frequency
Mucinous cystadenoma (sal. gl.)	+ *	-	-	-	unknown	+
Mucoepidermoid ca (sal. gl.)	+ *	-	*-*	*-/(* +*)*	*CRTC1::MAML2 #*	+ + +
Mucinous adenocarcinoma (sal. gl.)	-	-	*-*	*-/* +	*AKT1, TP53*	(+)
Mucinous carcinoma (skin)	-/+ *°	+	-/(+)	+ +	unknown	+
Endocrine mucin producing sweat gland carcinoma (skin)	-	-	+	+	unknown	(+)
Metastasis by mucinous carcinoma of other sites	-	-	-	-/+	depending on primary site	(+)

*Sal. gl*., salivary gland; *, positivity restricted to basal cells; °, restricted to in-situ component; # rarely *CRTC3::MAML2*

The most frequent mucinous tumor is salivary mucoepidermoid carcinoma. It represents the most frequent salivary carcinoma, located in 40% in minor glands. The prototypic presentation is a multi-cystic, less frequently solid proliferation with combined basaloid, intermediate, and glandular-luminal cytological differentiation and obligate, widely variable content of PAS-positive mucocytes (Part I: Fig. 3). Diagnosis of classical cases is usually straightforward, but there are many variants, most frequently with dominant or pure oncocytic, clear cell, squamous, or sclerosing phenotypes. In such difficult cases, the recurrent translocations *CRTC1::MAML2,* and less commonly *CRTC3::MAML2* (present in more than 90%), are helpful. The great majority are histologically low-grade with a favorable prognosis [2]. Mucoepidermoid carcinoma has not been established as a skin adnexal entity. However, hidradenocarcinoma carrying the same *CRTC1/3::MAML2* fusion can be indistinguishable from the clear cell variant of salivary mucoepidermoid carcinoma.

**Fig. 4:**
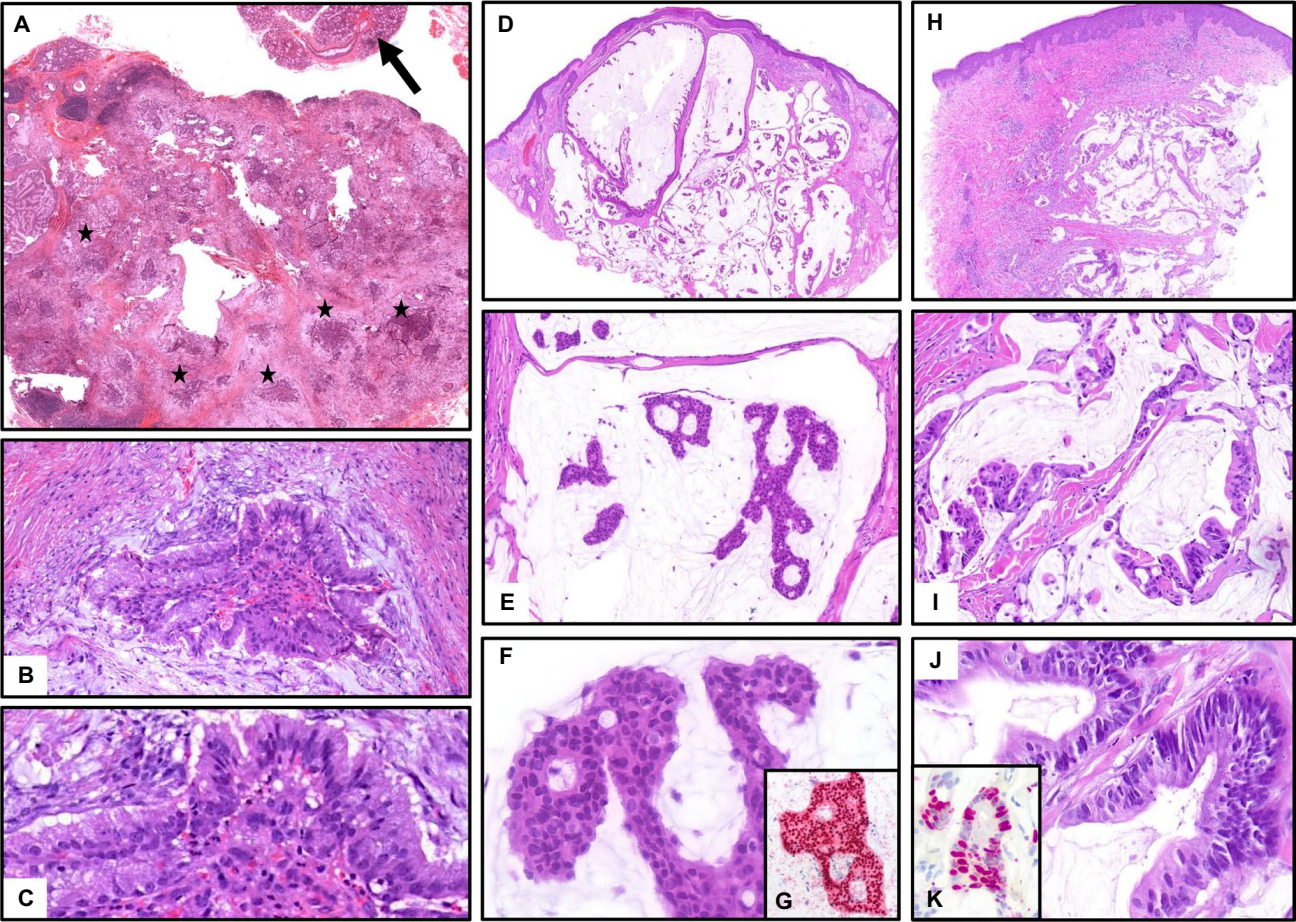
Mucinous carcinomas. **A**–**C** Well-differentiated mucinous adenocarcinoma of lip salivary gland with mutation of *AKT1*. **A** Overview with exuberant interstitial mucinous material with multiple “floating” islands of mucinous epithelium (stars). Arrow: minor salivary gland. **B**, **C** Uni-layered mucinous epithelium devoid of cellular atypia. **D**–**G** Mucinous carcinoma of the skin at the cheek. **D** Relatively well circumscribed lakes of mucin, separated by fibrous septa. **E**, **F** Strands of mucinous epithelium are floating in the mucin, no relevant cellular atypia. **G** Strong expression of the estrogen receptor. **H**–**K** Skin metastasis of mucinous carcinoma of the colon (CK20 positive). **H** Infiltrative lesion with lakes of extracellular mucin and irregular epithelium. **I**, **J** Extracellular mucin and highly atypical, multilayered glandular epithelium. **K** High proliferation (Ki67)

Mucinous adenocarcinoma of (mostly minor) salivary glands and cutaneous mucinous carcinoma represent somewhat corresponding tumor entities with analogous terminology; however, different (immune-)histology, molecular pathology, and prognosis exist. The very rare salivary mucinous adenocarcinoma probably (but still disputed) represents a spectrum from borderline mucinous neoplasia to low-grade and high-grade mucinous adenocarcinoma (Fig. 4) [15, 16]. The absence of a basal cell component and a recurrent *AKT1-E17K* mutation as a unifying genetic hallmark are important discriminators from mucoepidermoid carcinoma. Extracellular mucin, an additional mutation of the *TP53* gene, and poor prognosis are typical of high-grade salivary mucinous adenocarcinomas [15, 16] (Table 1).

In the skin, mucinous carcinoma shows a more uniform, usually well-circumscribed architecture with a honey-comb appearance of lakes of mucin with central adenoid, papillated, and solid aggregations of epithelial cells with little atypia. A facultative component of in situ carcinoma and obligate positivity for the estrogen receptor help to distinguish it from salivary mucinous adenocarcinoma and from cutaneous metastases from other sites (Fig. 4) [17, 18]. Local recurrence appears in up to 25%. Metastases, usually to lymph nodes, are rare. No recurrent genetic alterations have been described.

Endocrine mucin-producing sweat gland carcinoma is a rare low-grade carcinoma with a predilection for the eyelid [19] and expression of neuroendocrine markers. It consists of multiple solid and/or cystic nodules with small lakes of mucin. Immunohistochemically, synaptophysin, chromogranin, INSM1, BerEP4, CK7, and estrogen/progesteron receptors are expressed. There is a female predominance (67%) [20]. No recurrent genetic alterations have yet been described.

## Lymphadenoma and lymphoepithelial carcinoma

**Fig. 5:**
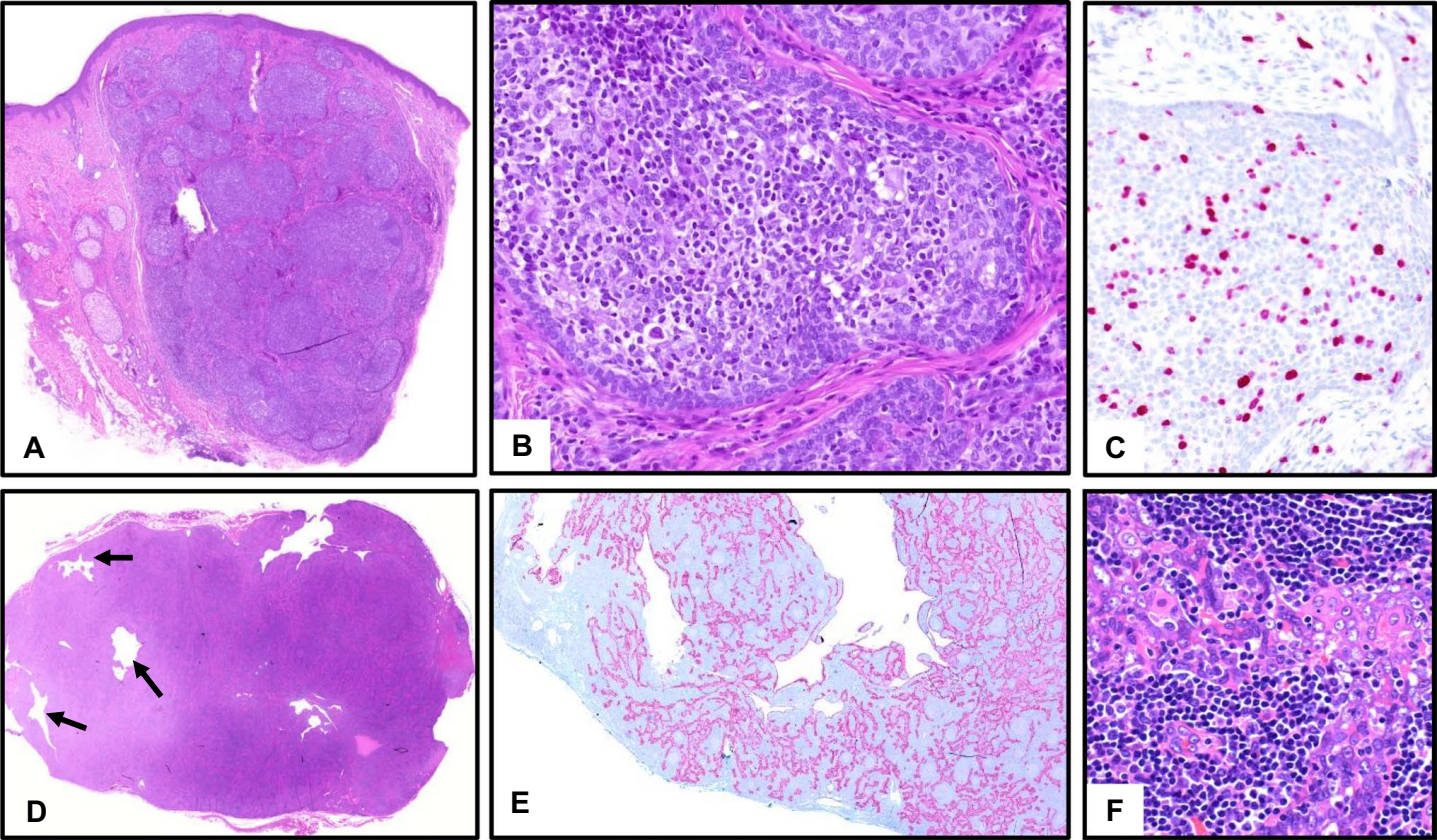
Lymphadenomas. **A**–**C** Formerly called “cutaneous lymphadenoma,” now termed adamantinoid trichoblastoma. **A** Well-circumscribed, lobulated dermal tumor. B Admixture of basaloid tumor formations with peripheral palisading and intermingled lymphocytes, no cellular atypia. Focal aggregations of plump fibroblasts (“germ-and-papilla” structures). **C **Low proliferation (Ki67, partly in lymphocytes). **D**–**F** Non-sebaceous lymphadenoma of parotid gland. **D** Well-circumscribed, cell-rich tumor with small cysts (arrows). **E** Netlike reticulated epithelium (red: CK14), in between reactive lymphocytic stroma (Haemalaun counter-stain: blue). **F** In high power, netlike strands of “lymphoepithelial” epithelium with exuberant intra- and inter-epithelial lymphocytes, no cellular atypia

In the salivary gland, lymphadenoma (sebaceous and non-sebaceous variants) is a well-circumscribed benign neoplasm with a glandular-cystic, basaloid, or lymphoepithelial proliferation within exuberant reactive lymphatic tissue, obviously developing in an intraparotid lymph node. There is no cellular atypia or increased proliferation (Fig. 5) [21, 22]. Very recently, our group proposed a continuum of salivary non-sebaceous lymphadenoma to thymus-like tumors with morphological and immunophenotypical features of thymic epithelial cells [23]. Analogous tumors (with or without thymus-like features) have not been reported in the skin.

In the skin, the term “cutaneous lymphadenoma” has historically been applied to a basophilic dermal neoplasm, peppered by lymphocytes. However, these tumors have now been identified as trichoblastic, consisting of follicular germinative cells (BerEP4 positive), often with “germ-and-papilla”-formation [24] and CK20-positive Merkel cells. The lymphocytic infiltration induces a pale appearance reminiscent of adamantinoma. Hence, the lesion has been renamed adamantinoid trichoblastoma (Fig. 5).

**Fig. 6:**
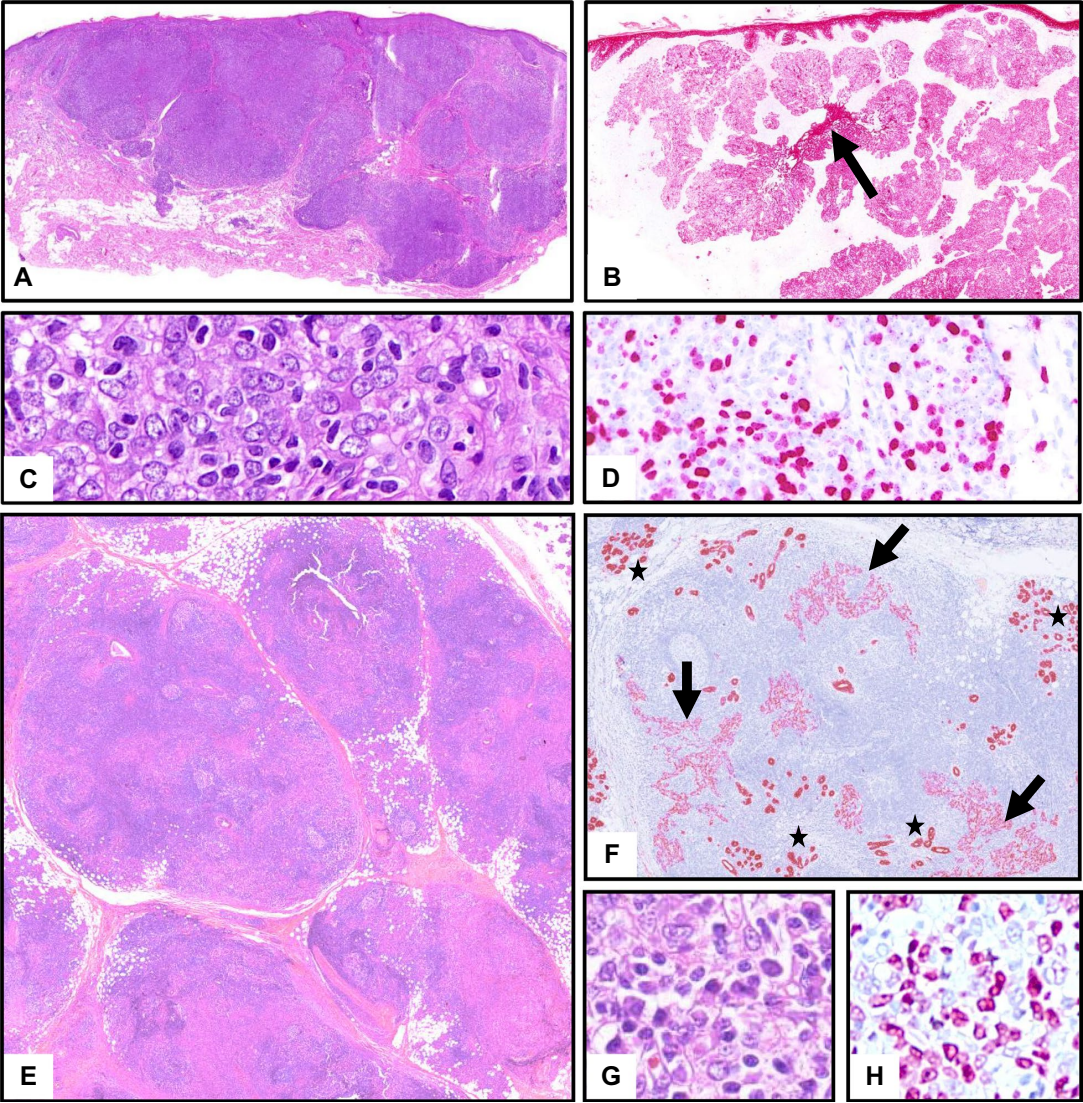
Lymphoepithelial carcinomas. **A**–**D** Cutaneous lymphoepithelial carcinoma (retroauricular). **A** Dermal, rather well-circumscribed, lobulated, cell-rich tumor. **B** Keratin-stain demonstrates lobulation, arrow indicates follicular structure, narrow “Grenzzone” to the epidermis. **C** High-power with admixture of ill-defined epithelial formations (large pale nuclei) with intermingled lymphocytes. **D** Strongly increased proliferation (Ki67), mainly in the epithelial component. **E**–**H** Lymphoepithelial carcinoma of parotid gland. E Largely preserved lobular architecture of salivary parenchyma (blue), irregularly infiltrated by carcinoma (red). **F** In keratin-stain remnants of salivary parenchyma (stars), in between infiltration by irregular carcinoma formations (arrows), in the background reactive lymphocytic stroma (blue; Haemalaun counter-stain). **G** High-power, reminiscent of cutaneous carcinoma (see **C**). **H** Strongly increased proliferation (Ki67)

The rare lymphoepithelial carcinomas of both organs are characterized by identical terminology and similar (immune-) histology, but by discrepant etiology and prognosis. Histopathology shows irregular, frequently inconspicuous islands of reticulated basaloid (“lymphoepithelial”) epithelium with many intraepithelial lymphocytes, embedded in reactive lymphoid tissue. Proliferative/mitotic activity is usually brisk. Cellular atypia is usually low in skin cases, varies in salivary cases from minor to severe [25]. While cutaneous tumors are relatively well-circumscribed, salivary (mostly parotid) tumors tend to be more infiltrative (Fig. 6). It is unclear whether cutaneous lymphoepithelial carcinoma is of epidermal or adnexal origin; it usually does not show connection to the epidermis.

Salivary lymphoepithelial carcinoma is in western, non-endemic areas characterized by positivity for EBV (EBER) in about half of the cases, by positivity for *TP53* in about 1/3 of cases, and by moderate prognosis. In contrast, cutaneous lymphoepithelial carcinoma is consistently negative for EBV and shows a favorable prognosis; metastases are extremely rare.

## Sebaceous adenoma and carcinoma

**Fig. 7:**
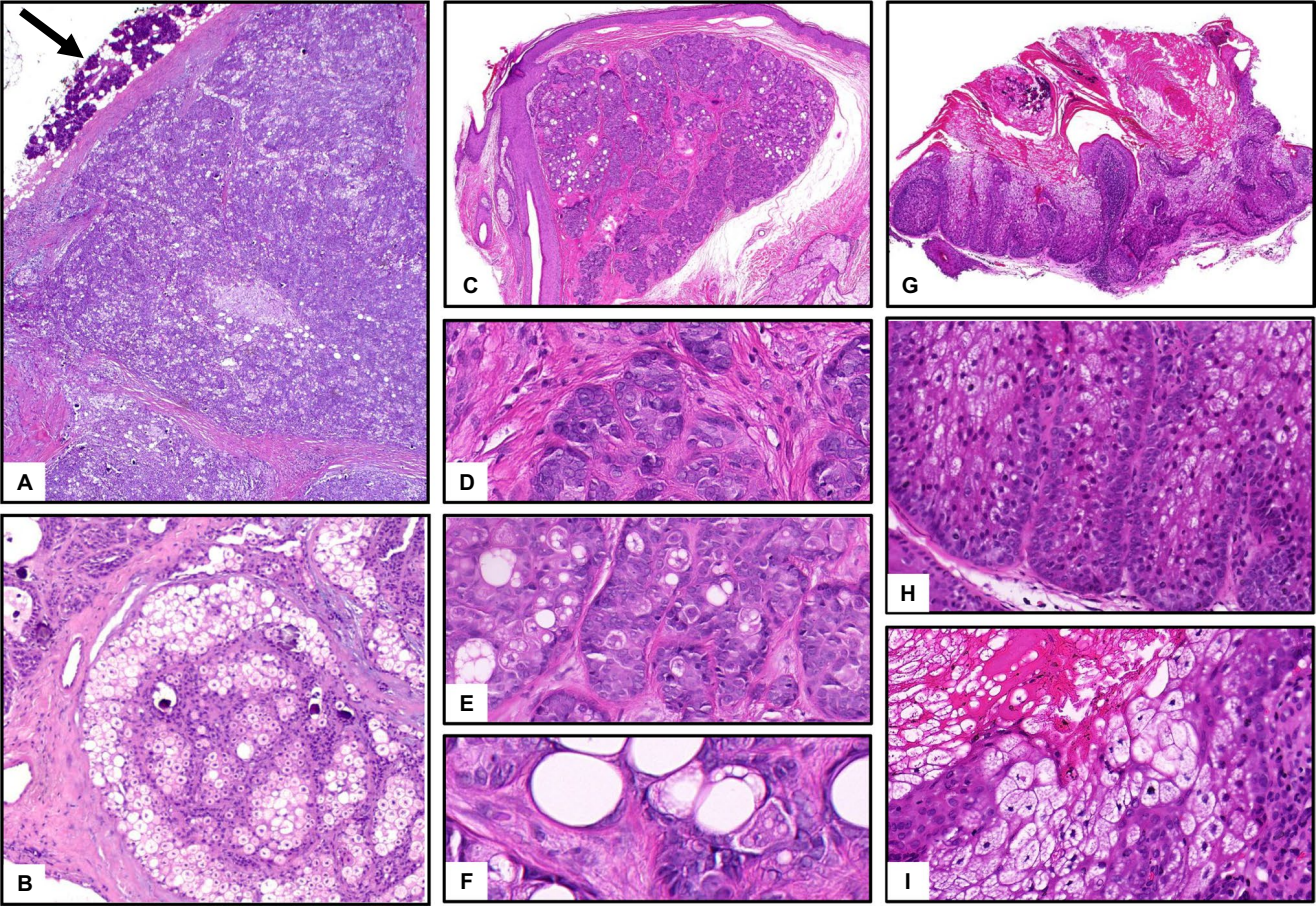
Sebaceous adenomas/tumors. **A**, **B** Parotid tumor with combined basaloid and sebaceous differentiation, devoid of atypia. Initial diagnosis: salivary sebaceous adenoma. Final diagnosis: sebaceous variant of epithelial-myoepithelial carcinoma with *HRAS*mut. **C**–**F** Cutaneous sebaceoma. Well circumscribed lesion with multinodular architecture and variable mixture of immature and mature sebocytes (**C**, **D**). Mature sebocytes are present as single cells or small collections, but not as large lobules (**E**, **F**). Vacuolated sebocytes may be uni- or paucivacuolar (**F**), as a phenocopy of embryonal development of sebocytes. **G**–**I** Cutaneous sebaceous adenoma. Irregularly shaped sebaceous lobules with large collections of vacuolated sebocytes connect horizontally to the epidermis; sebocytes show eosinophilic rather than clear cytoplasm and enlarged nuclei without scalloped borders (**H**); focal pagetoid pattern and relatively bland cytology (**I**)

Sebaceous tumors are much more common in the skin. Cutaneous sebaceoma presents as a solitary nodule most commonly on the face. It is well circumscribed, not connected to the epidermis, and consists of multiple, closely arranged nodules with a mixture of immature and mature sebocytes, sebaceous ducts, and cysts (Fig. 7C–F). Aggregations of mature sebocytes are small [24]. Immature sebocytes may include uni- or pauci-vacuolar cells and show large nuclei, but no overt nuclear atypia. Ripple pattern (wavy, parallel) or labyrinthian pattern (thickened basement membrane, cylindroma-like) is seen in immature variants.

**Fig. 8:**
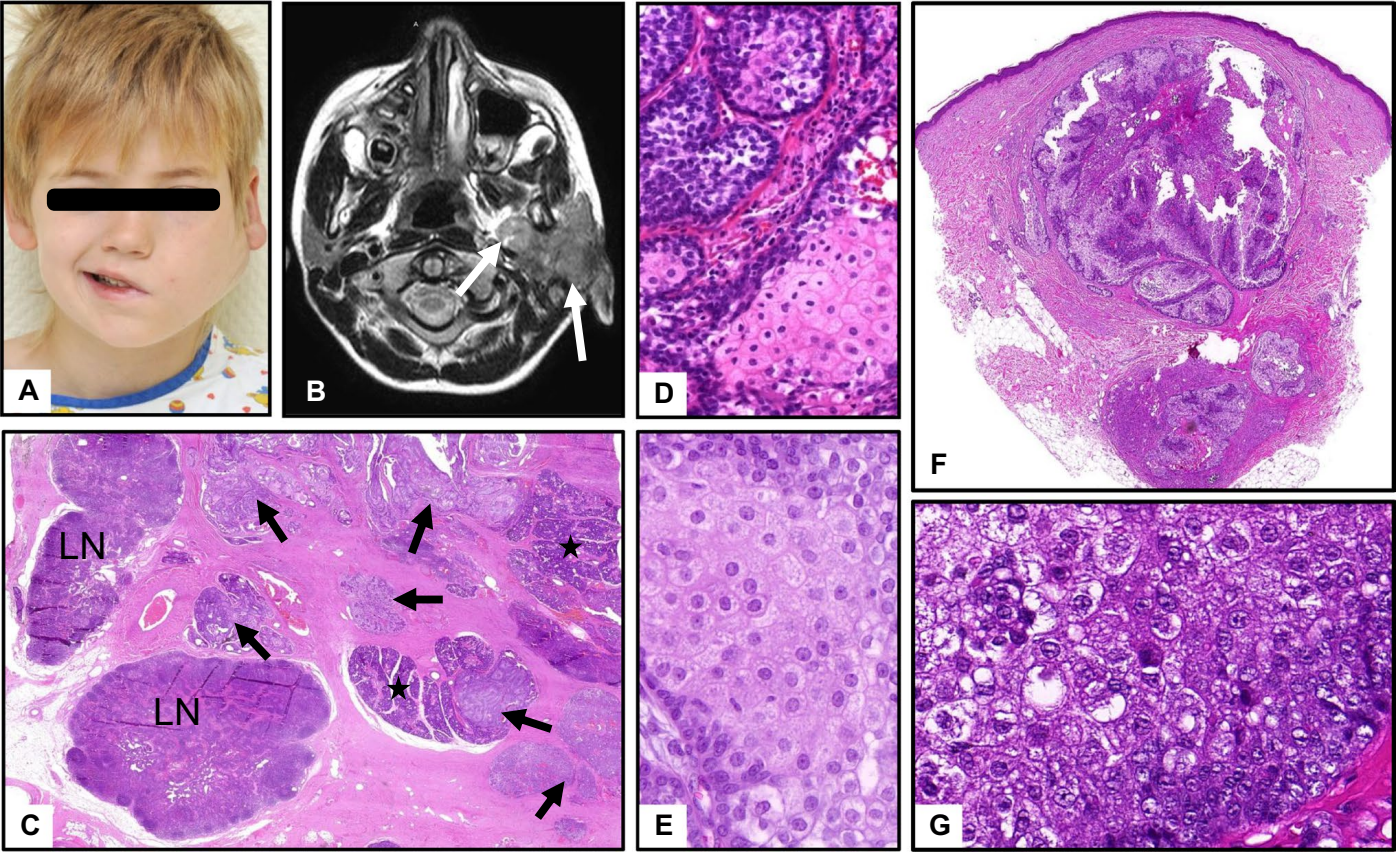
Sebaceous carcinomas. **A**–**E** Highly differentiated sebaceous carcinoma of the left parotid gland in a 7-year-old boy with palsy of the facial nerve (**A**) and large, infiltrative tumor (**B**, MRT, arrows). **C** Low-power HE with infiltrative tumor nodules (arrows) between residual salivary lobules (stars) and intraparotid lymph nodes (LN). **D**, **E** Basaloid proliferation with eosinophilic, partly clear cell, sebaceous differentiation, minor cellular atypia. **F**, **G** Cystic sebaceous carcinoma in a patient with Muir-Torre syndrome. Irregular, cystic proliferation with large lobules and infiltrative growth at the base, pleomorphism and crowding, enlarged nuclei without scalloped borders, prominent nucleoli and more eosinophilic cytoplasm, few necrotic cells

In contrast, cutaneous sebaceous adenoma (Fig. 7G–I) and well-differentiated sebaceous carcinoma (Fig. 8F, G), which probably represent a continuum, show quite different architecture with large, bizarre-shaped lobules of seemingly mature sebocytes, but often with eosinophilic, rather than clear, vacuolated cytoplasm, the latter with enlarged nuclei and atypical mitosis [26]. Lobules often connect to the epidermis with bowenoid in situ features and frequent ulceration. Cystic architecture is common (Fig. 8F). Poorly differentiated cutaneous sebaceous carcinoma shows a more immature, basophilic, crowded cytology, frequent atypical mitoses, and necrosis (Fig. 8G).

Both sebaceous adenoma and carcinoma of salivary glands are exceedingly rare. They histologically resemble their cutaneous counterparts with dominance of seemingly mature sebocytes, surrounded by basaloid cells with frequently enormously enhanced proliferation (Ki67), even in adenomas. Sebaceous carcinomas are characterized by blunt infiltration and necrosis. Figure 8 shows a very rare case of a cytologically bland appearing (“well differentiated”), however lethal sebaceous carcinoma in a 7-year-old child, where radiological and histological signs of infiltration are the only criteria of malignancy. However, the literature on salivary sebaceous adenocarcinoma is rather inconclusive, given that the majority of putative salivary sebaceous carcinomas seen in consultation centers are indeed reclassified as sebaceous variants of epithelial–myoepithelial carcinomas (Fig. 7A, B), so that the exact frequency of genuine sebaceous adenocarcinoma is unclear. Likewise, salivary sebaceous adenoma is exceedingly rare and the possible relationship to bland (low-risk) epithelial–myoepithelial carcinomas with extensive sebaceous features remains to be verified.

Putative sebaceous differentiation can be highlighted immunohistochemically with Adipophilin and PRAME [26]. Cutaneous (but not salivary) sebaceous adenomas and carcinomas are associated with Muir-Torre syndrome (OMIM **#** 158,320), caused by a heterozygous mutation in the MSH2 (chromosome 2p21–p16) or MLH1 gene (chromosome 3p22). Patients are at risk for internal malignancies (colorectal, endometrial, urologic, and upper gastrointestinal neoplasms). In Muir-Torre syndrome, sebaceous adenomas and carcinomas (but not sebaceomas) typically appear as multiple lesions on the face. Loss of expression of MSH2, MSH6, or MLH1 can be demonstrated immunohistochemically. Chronic sun exposure and papilloma virus may contribute to development in cases without syndromic association.

In the skin, prognosis for small, well-differentiated sebaceous carcinoma is favorable if the lesion is removed completely. Apart from that, sebaceous carcinomas are potentially aggressive tumors with a tendency to recur and metastasize, most frequently to lymph nodes [27]. It is important to emphasize that sebaceous carcinoma of the eyelid is a totally separate entity with much higher mortality and no association to Muir-Torre syndrome [27].

## Microcystic adnexal carcinoma versus (salivary) sclerosing microcystic adenocarcinoma

**Fig. 9:**
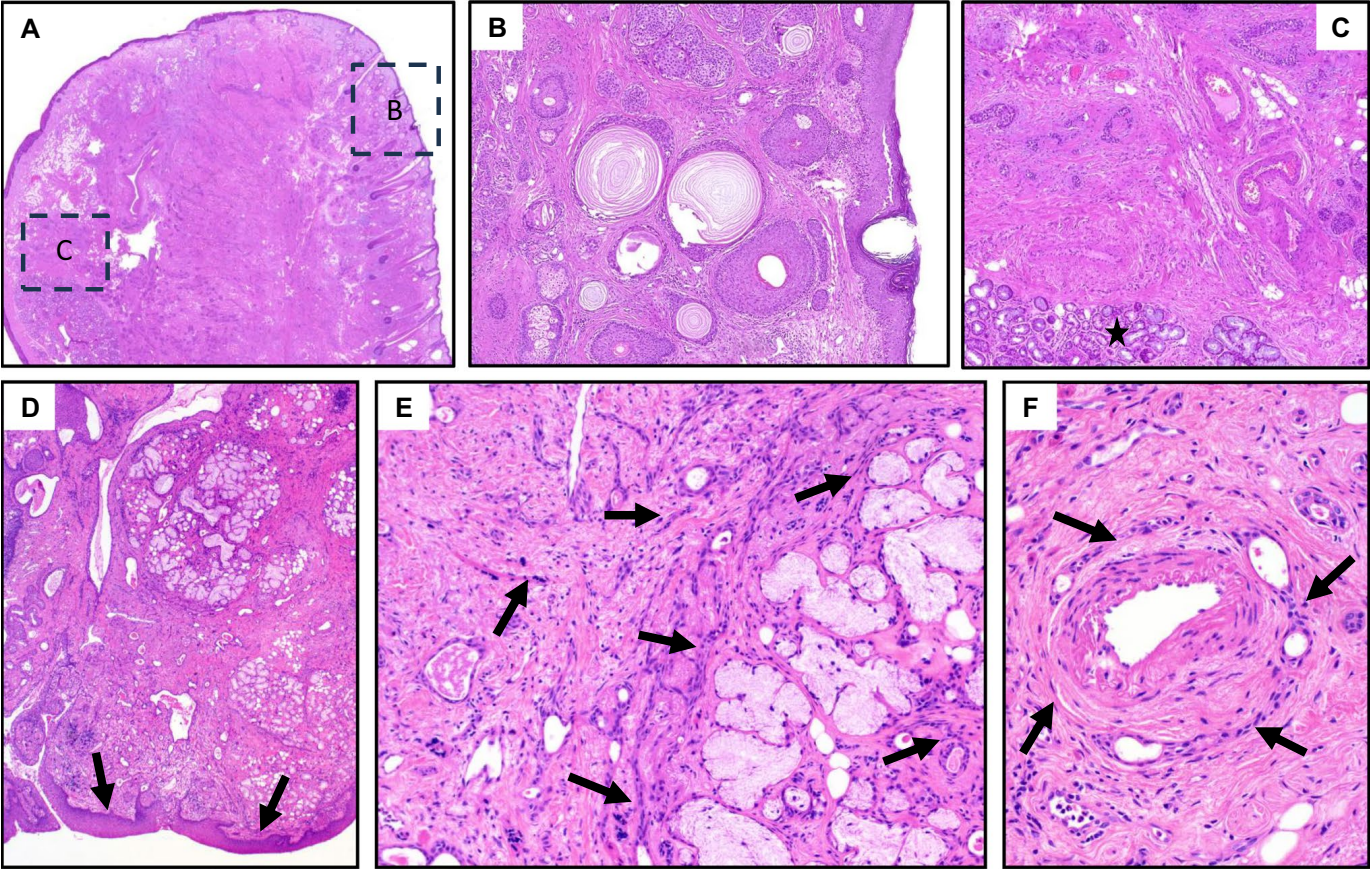
**A**–**C** Cutaneous microcystic adnexal carcinoma in the lower lip. **A** Massive, scar-like enlargement of the lower lip (skin: right, mucosa: left). **B** Magnification of the marked area in A, here with dominance of microcystic squamous differentiation, a hallmark of this carcinoma. **C** Magnification of the marked area in **A**, here with dominance of bluntly infiltrative epithelial strands without cellular atypia, within massive desmoplastic sclerosis. Minor lip gland (bottom, star). **D**–**F** Sclerosing microcystic adenocarcinoma of a palatal minor salivary gland. **D** Diffuse scar-like sclerosis, engulfing salivary lobules and with cystic dilatation of salivary ducts (arrows: palatal mucosa). **E**, **F** Irregular, netlike narrow strands of cytologically bland basaloid epithelium (arrows), partly bilayered and with small cysts, engulfing salivary lobules and vessels and inducing major sclerosis with minimal inflammation.

Both carcinoma types are similar with respect to analogous terminology, (immuno-) histology, and prognosis. They show a similar, grossly infiltrative pattern with inconspicuous tumor strands, accompanied by extensive sclerosis, mimicking a scar. The stereotypical presentation in the skin is cornifying cysts in the upper part, small solid aggregations in the middle part, and long tubules in the lower part (Fig. 9A–C) [24]. Typical sites are the nasolabial area, upper lip, but also the periorbital area, ear, and scalp, presenting with a slowly growing, indurated area, sometimes with a burning sensation and numbness due to neurotropism. A similar scar-like sclerosis may be seen in squamoid eccrine ductal carcinoma and sclerosing basal cell carcinoma.

A strikingly similar growth pattern characterizes the recently established, obviously very rare entity of sclerosing microcystic adenocarcinoma of salivary glands, so far only described in minor glands, obviously with favorable prognosis (Fig. 9D–F) [28]. Consistent molecular alterations have not been described in both organs.

## Final synoptic comparison of tumors of both organs

Table 2 summarizes a general comparison of basic principles of benign and malignant salivary gland and skin adnexal neoplasms. This condensed comparison inevitably is full of simplifications, exceptions, and contradictions. Important aspects are reconsidered in more detail in the following paragraphs.

**Table 2:** Simplified synoptic comparison of general aspects of salivary gland and cutaneous adnexal tumors (combined for all benign and malignant tumors)

	**Salivary gland tumors**	**Cutaneous adnexal tumors**
Number of tumor entities	Great (*n* = 36)	Enormous (*n* = 47)
Differentiation	Many salivary tumor entities are similar to …	… adnexal tumors of eccrine, apocrine, and sebaceous, but not follicular differentiation
Relative frequency of malignant tumors	About 30%*	By far less common than benign tumors (exact data not available) #
Diagnostic problems	Distinction of benign and low-grade malignant tumors	Superficial curettage/shave (incomplete architecture)
Importance of immunohistology	Frequently high	Generally low
Importance of diagnostic molecular pathology	High	In general low, but increasing
Surgical resection	Frequently difficult	Usually feasible
Recurrences	Rather frequent	Rare (if operated appropriately)
Prognosis (malignant tumors)	On average moderate	On average favorable
Multifocality/involvement in syndromes	Both aspects very rare #	Both aspects more common

*24.1% in tumors of major glands and 54.1% in minor glands [29]; # basal cell carcinoma excluded, # with exclusion of Whartin tumor (multifocality)

### Principal lack of differentiation patterns

Due to the absence of a follicular differentiation in salivary anatomy, tumors with a component of follicular differentiation are purely cutaneous type and are absent in salivary glands. As both organs show identical peripheral acinar differentiation, it is totally unclear why the only tumor entity with acinar differentiation (acinar cell carcinoma) is restricted to salivary glands. The situation is similar to oncocytic differentiation, which is also largely restricted to salivary glands. Rare cutaneous oncocytoma has usually been reported in the periocular location, where it presumably represents lacrimal glandular, rather than cutaneous derivation. Occasionally, oncocytic cells have been observed as metaplasia in cutaneous hidradenoma. However, true oncocytic differentiation must be differentiated from oncocyte-like appearance, which can be seen in a broad variety of cell types.

### Diagnostic relevance of immunohistochemistry

Overall, the diagnostic impact of immunohistochemistry is regarded to be much more helpful in salivary tumors, the reasons for that difference being complex and speculative. Presence or absence of biphasic-tubular structures (CK18/7 versus p63, CK14) is of paramount importance in the differential diagnosis of salivary tumors, obviously less so in cutaneous tumors. The distinction of tumors with dominant basal versus myoepithelial differentiation is limited in both organs due to major variation of the so-called myoepithelial markers. The value of proliferative markers (Ki67) is limited in both organs due to usually low proliferation both in benign and low-grade malignant tumors [29]. Considering the obligate absence of follicular differentiation in salivary tumors, presence versus absence of follicular markers can be helpful (BerEP4, PHLDA1, Cytokeratin 17). Adipophilin and PRAME may help to confirm sebaceous differentiation.

Immunohistological markers specific for certain tumor entities (like DOG-1 and NR4A3 in salivary acinic cell carcinoma) are very rare. Positivity for estrogen/progesteron receptors is quite specific for cutaneous mucinous carcinoma, while positivity for the androgen receptor is highly characteristic for salivary duct carcinoma and carcinoma ex pleomorphic adenoma, and is typical for a series of apocrine type cutaneous adnexal tumors. Nuclear positivity for beta-Catenin is present (and in this case specific) for part of salivary basal cell adenoma and carcinoma. Neuroendocrine markers are expressed in endocrine mucin producing sweat gland carcinoma and in very rare salivary neuroendocrine carcinomas [19]. Immunohistochemistry is generally very helpful for the evaluation of putative metastases, manifesting in the skin or in salivary glands.

### Diagnostic relevance of molecular pathology

Table 3 summarizes consistent molecular alterations of the pairs of related tumors presented here, exhibiting more frequently identical and more rarely discrepant genetic alterations. Vice versa, the presence of the same genetic alteration in two principally different tumors is rare (for example: *CRTC1::MAML2* in salivary mucoepidermoid carcinoma versus in cutaneous hidradenoma). The major diagnostic value of the genetic alterations (especially in salivary tumors) is based on these facts: The great majority of genetic alterations (predominantly translocations) are specific and exclusive for many tumor entities and the frequency is mostly high (between 50 and almost 100%).

**Table 3:** Recurrent molecular alterations in related tumor pairs of both organs (restricted to most frequent mutations/variants; identical molecular alterations in bold type)

**Salivary tumors**		**Adnexal tumors**	
Pleomorphic adenoma/myoepithelioma	***PLAG1* rearr,* HMGA2* rearr**	Apocrine mixed tumor/myoepithelioma	***PLAG1* rearr*, HMGA2* rearr**
Basal cell adenoma	*CTNNB1-I* 35 T, ***CYLD* mut**	Spiradenoma/cylindroma	*ALPK1-*V1092A, ***CYLD* mut**
Secretory carcinoma	***ETV6::NTRK3***	Secretory carcinoma	***ETV6::NTRK3***
Microsecretory carcinoma	***MEF2C::SS18***	Microsecretory carcinoma	***MEF2C::SS18***
Sialadenoma papilliferum	***BRAF-V600E***	Syringocystadenoma papilliferum	***BRAF-V600E***
Adenoidcystic carcinoma	***MYB::NFIB***	Adenoidcystic carcinoma	***MYB::NFIB***
Mucinous adenocarcinoma	*AKT1*-E17K*, TP53*mut	Mucinous carcinoma	-
Sebaceous carcinoma	-	Sebaceous carcinoma	*MLH1, MSH2, or MSH6* mut

Overall, the diagnostic impact of testing for mutations/translocations, including the new technique of DNA-methylation [30], is well established for difficult salivary tumors and here is regarded to be very high [5, 29, 31]. Especially, the distinction between several benign and low-grade malignant entities of salivary glands relies not infrequently on additional molecular methods (see Figs. 1, 3, and 4, as well in part I: Figs. 3 and 4). On the other hand, the relevance of molecular techniques for the diagnosis of cutaneous adnexal tumors is lower; however, it is as well increasing. Molecular confirmation is usually not required in benign cutaneous adnexal tumors. Generally, the still limited availability of specialized molecular testing and the costs are limiting factors, probably more so in dermatohistology.

### The principle of intraductal neoplasia

The pathogenetic principle of intraductal neoplasia (“dysplasia,” carcinoma in situ) does principally exist in both organs; however, it is much less frequent and has much less diagnostic and prognostic impact than in many other organs (for example, breast or prostate). Intraductal neoplasia in salivary tumors exists in salivary duct carcinoma and in carcinoma ex pleomorphic adenoma, in cutaneous tumors in mucinous adenocarcinoma [18] and in a variety of benign tumors with initial malignant transformation [32]. Cases of pure intraductal carcinoma (without an invasive component) are exceedingly rare in both organs.

### Grading of malignancy and high-grade malignant transformation

For many reasons, conventional grading of malignancy in salivary and cutaneous adnexal tumors is restricted to very few entities; for example, in salivary glands, to mucoepidermoid carcinoma [2]. In the great majority, salivary and cutaneous malignant tumor entities are by definition either low-grade or, more rarely, high-grade malignancies. Many entities are so rare that the establishment of criteria like low- versus high-grade generally is not possible due to a lack of follow-up data. Both secondary malignant transformation ex benign tumors (in both organs not infrequent) and high-grade transformation ex different types of low-grade carcinomas (relatively frequent in salivary carcinomas) usually represent high-grade carcinoma per definition.

### Overlaps between several organs are underrecognized

The herein presented overlap of analogous tumors is not restricted to salivary glands and cutaneous adnexa. The mammary gland comprises so-called salivary analogous tumors of both ductal (e.g. secretory carcinoma) and biphasic–myoepithelial differentiation (e.g. fibroadenoma, phylloides tumor). Odontogenic tumors as well comprise overlap with respect to ghost cell tumors and to intraosseous mucoepidermoid and clear cell carcinoma. A more detailed investigation of these overlaps may enhance our scientific understanding of these tumors and may help in difficult differential diagnoses in overlapping topographical areas.
